# Beta power as a neural correlate of sensory features in autistic individuals

**DOI:** 10.1186/s11689-026-09685-1

**Published:** 2026-03-18

**Authors:** Julie Chaudet, Julien Pichot, Amandine Pedoux, Mathis Fleury, Anna Maruani, Valérie Vantalon, Elise Humeau, Thomas Bourgeron, Josselin Houenou, Guillaume Dumas, Edouard Duchesnay, Richard Delorme, Anton Iftimovici, Aline Lefebvre

**Affiliations:** 1 PsyBrain Team, UNIACT Unit, NeuroSpin, CEA Paris-Saclay, Gif-Sur-Yvette, France; 2 GAIA Team, BAOBAB Unit, NeuroSpin, CEA Paris-Saclay, Gif-Sur-Yvette, France; 3 Institut Pasteur, Université Paris Cité, ‘Human Genetics and Cognitive Functions’ Team, CNRS UMR 3571, Paris, France; 4 Child and Adolescent Psychiatry Department, Robert Debré University Hospital, APHP, and Université Paris Cité, Paris, France; 5 Department of Psychiatry, Faculty of Medicine, Mondor University Hospital, APHP, ‘Translational Psychiatry’ Team 15, INSERM U955, Créteil, France; 6 Department of Psychiatry, Faculty of Medicine, CHU Sainte-Justine Research Centre, ‘Precision Psychiatry and Social Physiology’ Team, Université de Montréal, Montréal, QC Canada; 7 Institute of Psychiatry and Neuroscience of Paris (IPNP), Université Paris Cité, INSERM U1266, ‘Pathophysiology of psychiatric disorders’ Team, CNRS GDR 3557, Paris, France; 8 GHU Paris Psychiatrie et Neurosciences, Sainte Anne Hospital, APHP, Paris, France; 9 Faculty of Medicine, Université Paris-Saclay, Kremlin-Bicêtre, France; 10 Neurodevelopmental Disorders Advice and Expertise Service, Fondation Vallée, Gentilly, France

**Keywords:** EEG, ASD, Power, Sensory processing, Restricted and repetitive behavior

## Abstract

**Background:**

Autism spectrum disorder (ASD) is characterized by social communication difficulties, restricted and repetitive behaviors, and sensory atypicalities. Its pathophysiology remains poorly understood, and no diagnostic biomarker is currently available. However, studies support the hypothesis of an imbalance in the neuronal excitation/inhibition (E/I) balance. Beta and gamma oscillatory powers are described in the literature as indirect electrophysiological features reflecting network dynamics related to this balance. This study takes a dimensional approach to investigate whether alterations in these rhythms are linked to the severity of autistic symptoms.

**Methods:**

A total of 127 individuals with ASD, including a sensory-assessed subgroup of 57 participants, all aged 5 to 17 years, underwent resting-state high-density electroencephalography (HD-EEG) recordings with eyes closed. Absolute power was extracted from the beta and gamma frequency bands for six regions of interest. Multiple linear regression models were used to investigate the relationships between beta and gamma powers and the clinical dimensions of ASD, while controlling for the effects of age and sex.

**Results:**

Beta power was positively correlated with sensory hyposensitivity across all regions (all corrected *p*<.02, *n* = 57) and with sensory hypersensitivity in the central, parietal, and left temporal regions (all corrected *p*<.05, *n* = 57). In addition, an exploratory result suggested that an increase in gamma power may be associated with the severity of restricted and repetitive behaviors (RRBs) in the right temporal region (uncorrected *p*=.01, *n* = 127).

**Conclusions:**

These innovative results call for further analysis, including an investigation of other electrophysiological markers providing indirect profiles of E/I balance. Nevertheless, they open new avenues for a better understanding of the neurobiological processes and early diagnosis of ASD.

**Supplementary Information:**

The online version contains supplementary material available at 10.1186/s11689-026-09685-1.

## Introduction

Autism spectrum disorder (ASD) is a neurodevelopmental condition characterized by impaired social communication, restricted and repetitive behaviors (RRBs) and sensory atypicalities [1]. Its prevalence is 1–2%, making it a major societal challenge [2]. However, its aetiology and pathophysiology remain poorly understood. This difficulty is mainly due to the observed clinical, biological, and environmental heterogeneity, as well as the complex gene-environment interactions underlying ASD [3, 4]. Despite these challenges, several pathophysiological mechanisms have been implicated in ASD [5]. Among these, one mechanism supports the hypothesis of an imbalance in the neuronal excitation/inhibition (E/I) balance [6], characterized by a disruption between excitatory glutamatergic neurotransmission and inhibitory GABAergic neurotransmission [7, 8].

Rubenstein and Merzenich [6] initially proposed this E/I imbalance because of the high prevalence of epilepsy in ASD. This imbalance may be associated with an increased E/I ratio in individuals with ASD, leading to hyperexcitability of cortical circuits. Currently, one of the key challenges in understanding this balance and its implications in ASD is the development of measures to study it.

Electroencephalography (EEG) offers many advantages for deriving markers of the E/I balance. It is a noninvasive technique with excellent temporal resolution, low cost and easy accessibility for routine clinical use. In addition, the use of high-density EEG (HD-EEG) may increase spatial resolution through the use of source localization techniques. EEG markers show great potential in research on psychiatric disorders [9], particularly in ASD [10, 11]. The development of this technique could not only enhance our understanding of the associated neurobiological mechanisms but also enable earlier diagnosis.

Beta and gamma powers have been described in the literature as indirect electrophysiological features reflecting network dynamics related to the E/I balance [12, 13]. Beta and gamma oscillations result from the coordination between the excitation of cortical pyramidal neurons and the regulation of this excitation by inhibitory interneurons, which use an inhibitory feedback mechanism to maintain the balance of electrical activity in the brain. Importantly, all oscillatory activity measured by EEG, regardless of frequency band, results from reciprocal interactions between excitatory and inhibitory neurons. Nevertheless, beta and gamma bands have been more extensively investigated than other frequency ranges in preclinical and pharmacological studies, providing a better understanding of how excitatory and inhibitory neurons contribute to the cellular and circuit mechanisms underlying their modulation [14, 15]. However, the existing literature remains highly heterogeneous, making it difficult to assert that an increase in beta or gamma power is specifically linked to a rise in excitation or inhibition. Throughout this study, references to the E/I balance are therefore interpreted as indirect reflections of network-level dynamics inferred from oscillatory activity, rather than direct or direction-specific measures of synaptic excitation or inhibition.

Beta oscillations (12–30 Hz) are associated with alertness, cognitive control, and sensorimotor integration [16, 17]. This range allows us to take into account the low beta range (12–20 Hz), which includes the sensorimotor rhythm (12–15 Hz) [18–20]. Gamma waves, with a frequency range of 30–100 Hz, are associated with complex cognitive processes, information processing, memory and sensory perception [21]. The involvement of these oscillations in such functions is particularly relevant to the symptoms observed in ASD, and alterations in these rhythms have indeed been reported in this condition [11, 21, 22]. However, the direction of these changes and their relationship with autistic symptomatology remain unclear and are subject to nuanced findings.

ASD presents significant symptomatic heterogeneity, making it difficult to identify common neurobiological mechanisms [23–25]. Consistent with current trends in ASD research, we adopted a dimensional framework within the ASD group to capture intra-group variability, allowing us to study continuous variations in symptoms and EEG markers rather than relying on categorical case-control differences. This would allow us to study different neurophysiological profiles by linking EEG biomarkers, as indirect reflections of the E/I balance, with the diversity of clinical manifestations.

Based on the existing literature, we consider beta and gamma oscillatory powers as indirect reflections of the E/I imbalance. Therefore, this study aims to investigate how within-group variations in these rhythms may be linked to the severity of autistic symptomatology.

## Methods

### Participants

All participants were recruited as part of the SoNeTAA (Social Neuroscience for Therapeutic Approaches in Autism) platform, developed within the Child Psychiatry Unit of the Robert Debré Hospital (APHP, Paris, France) in partnership with the Institut Pasteur (https://research.pasteur.fr/en/project/sonetaa/*).* A total of 127 participants with ASD (IQ mean = 101.3, SD = 18.2), aged 5 to 17 years (mean = 10.26, SD = 2.84; female/male = 17/110) were included (Table 1). Participants were diagnosed according to the DSM-5 criteria [1] and were assessed using the Autism Diagnostic Interview Revised (ADI-R) [26], the Autism Diagnostic Observation Schedule (ADOS-2) [27] and clinical reports from experts in the field, who made the final diagnostic decision. Some participants who were under medication were included in the study, their medication details are provided in Supplementary Table S1-S2. All the data were collected using a numerical identifier, and personal health information was maintained solely by the data collection site.

**Table 1:** Participants descriptive and clinical information

Group category	Clinical measures	*N*	Age (years) (Mean/SD)	Sex (F/M)	Score (Mean/SD)
Complete group	ADOS-2 CSS	127	10.3 (2.8)	17/110	6.5 (2.8)
	ADI-R A				14 (6.5)
	ADI-R B				10 (5.0)
	ADI-R C				4.9 (2.7)
SRS-assessed subgroup	SRS-2 T-score				
	*Social motivation*	113	10.4 (2.8)	15/98	69.7 (12.8)
	*Social communication*				72.7 (11.7)
	*Social awareness*				67.6 (12.3)
	*Social cognition*				68.6 (12.6)
	*Total score*	116	10.3 (2.8)	16/100	73 (11.4)
Sensory- assessed subgroup	SSP Hyper	57	10.2 (2.9)	8/49	2.4 (0.9)
	SSP Hypo				2.4 (0.7)

*Abbreviations: N* Number of participants, *F* Female, *M* Male, *ADOS-2 CSS* Autism Diagnostic Observation Schedule—second version—Calibrated Severity Score, *ADI-R* Autism Diagnostic Interview Revised, *ADI-R A* ADI-R Social Interaction domain score, *ADI-R B* ADI-R Communication domain score, *ADI-R C* Stereotypes & Restricted Interests domain score, *SRS-2 T-score* Social Responsiveness Scale, Second Edition, Standardized T-score, *SSP* Short Sensory Profile, Hyper-Hyposensory scores

Complete group: all participants in the sample; SRS-assessed subgroup: participants who completed the SRS assessment, with corresponding scores available; Sensory-assessed subgroup: participants who completed the SSP assessment, with corresponding scores available

The full sample in this study was referred to as the ‘Complete group’, while the subset of participants who underwent the Responsiveness Scale - Second Edition (SRS-2) [28] assessment formed the ‘SRS-assessed subgroup’, and those who completed the sensory evaluation using the Short Sensory Profile (SSP) [29] were designated as the ‘Sensory-assessed subgroup’ (Table 1).

### Clinical phenotypes

To assess the relationship between autistic symptomatology and the power of beta and gamma oscillations, we used several standardized clinical scales commonly used in ASD, as described in Table 2.

**Table 2:** Overview of the different clinical assessment measures

Clinical measures	Description
Social Responsiveness Scale, Second Edition (SRS-2) [28]	A 65 items hetero-questionnaire using a 4-point Likert scale, designed to screen for ASD. It includes five subdomains: Social Awareness, Social Cognition, Social Communication, Social Motivation, and Restricted Interests and Repetitive Behavior.
Autism Diagnostic Observation Schedule, Second Edition (ADOS-2) [27]	A semi-structured diagnostic assessment tool for ASD that evaluates social communication and stereotyped behaviors. Given that different modules were administered depending on participants’ age and language level, we used the Calibrated Severity Score (CSS) to enable standardized comparisons across modules.
Autism Diagnostic Interview-Revised (ADI-R) [26]	A structured hetero-questionnaire with 93 items for the diagnosis of ASD. The ADI-R includes three subdomains: Reciprocal Social Interactions (ADI-R A), Communication and Language (ADI-R B), and Stereotyped and Repetitive Behaviors (ADI-R C).
Short Sensory Profile (SSP) [29]	A 38-items hetero-questionnaire assessing sensory peculiarities using a 5-point Likert scale. In this study, the items of the SSP have been divided into two distinct subscores, grouping items related to hyper- or hyposensory responses.

Overall symptomatology was assessed using the ADOS-2 Calibrated Severity Score (CSS) (*n* = 127, mean age = 10.3, female/male = 17/110) and the total T-score from the SRS-2 (*n* = 116, 11 missing data, mean age = 10.3, female/male = 16/100) (Table 1).

We defined the social communication domain using the ADI-R subdomains A (Reciprocal Social Interaction) and B (Communication) (*n* = 127, mean age = 10.3, female/male = 17/110), as well as the Social Communication, Social Motivation, Social Awareness and Social Cognition subscales T-scores from the SRS-2 (*n* = 113, 14 missing data, mean age = 10.4, female/male = 15/98) (Table 1).

The restricted and repetitive behaviors and sensory symptoms domains was represented by the ADI-R subdomain C (Restricted, Repetitive, and Stereotyped Patterns of Behavior), the corresponding RRB subscales of the SRS-2 (*n* = 113, 14 missing data, mean age = 10.4, female/male = 15/98), and the SSP, which was available for a subsample of 57 participants (mean age = 10.2, female/male = 8/49) (Table 1).

To explore hyper- and hyposensitivity aspects, and given the lack of a scale providing direct access to these scores across sensory domains, we decided to calculate the hyper- and hyposensory scores from the SSP, the only scale available in our dataset. For better readability of the results, the SSP item scores (ranging from 1 to 5) were reversed, with 1 = never and 5 = always. We calculated hyper- and hyposensory scores by following the method described by Lefebvre et al. [30]. In this approach, the 38 SSP items are divided into two distinct subscores: one grouping items related to hypersensitivity (*n* = 19) and the other to hyposensitivity (*n* = 16) and 3 uncertain items are excluded (Supplementary Table S3).

### EEG recording

All subjects were recorded in resting-state with eyes open and closed for periods of 3 to 5 min using a 128-GSN hydrocel Geodesic montage. In this study, we restricted our analysis to the eyes-closed condition in order to maximize resting-state signal stability by minimizing visual stimulation. Sampling was performed at 1000 Hz, with the reference electrode at the Cz position.

### Pre-processing and processing EEG data

Signal analysis was performed entirely in python using MNE-python [31]. The recordings were concatenated to retain only the closed-eye segments (mean duration = 95.94 s, SD = 13 s, range = 60–165 s), then rereferenced to the mean and filtered between 0.1 and 45 Hz. Edge effects due to concatenation and filtering were removed. Then, electrodes located outside the scalp, mainly facial electrodes, were automatically removed due to high levels of artifacts related to muscle activity, sweat, and eye movements (E14, E17, E21, E48, E119, E126, E127; *n* = 7). The recordings were then visually inspected by two trained raters, experienced in artifact recognition and EEG interpretation, to remove any remaining artifacts. Visual inspection remains interesting for ensuring data quality, particularly in developmental or clinical populations where automatic algorithms may fail to capture atypical noise patterns [32].

The absolute power spectral density (PSD, expressed in µV²/Hz) of the beta (12–30 Hz) and gamma (30–45 Hz) bands was computed for each electrode using Welch’s method (2-second Hann window with a 50% overlap, and a spectral resolution of 0.5 Hz). PSD values were then averaged across electrodes within six predefined regions of interest (ROIs) corresponding to lobular scalp-level averages : frontal, central, parietal, occipital, left temporal and right temporal for each frequency band (Supplementary Figure S1). The temporal lobe was divided into right and left hemispheres to better account for hemispheric asymmetries observed in this region, particularly in ASD [33].

### Statistics

The relationships between clinical variables and beta and gamma PSD for each ROI were explored using multiple linear regression models to control for the effects of age and sex. Age was modeled as a continuous covariate in all regression models to account for developmental changes in EEG power across the 5–17 year age range. Homoscedasticity of residuals was assessed for each model using the Breusch-Pagan test and by visual inspection of residual-fitted plots (Supplementary Figures S2–S4). In cases where heteroscedasticity was detected, heteroscedasticity-consistent standard errors (HC3) were applied in the model. We also assessed the influence of individual observations using Cook’s distance (Supplementary Figures S5–S7, Supplementary Table S4). To control for multiple comparisons, False Discovery Rate (FDR) correction was applied separately for each combination of frequency band (beta, gamma) and clinical measure (e.g., ADOS-2 CSS, ADI-R domains, sensory scores). Within each combination, the six p-values corresponding to the six ROIs were corrected together. The *p*-values are considered significant for *p*_*corr*_ <0.05. Cohen’s *f* ^2^ was used to calculate effect sizes for the multiple linear regression models, with thresholds for small, medium, and large effects defined as *f* ^2^≥0.02, *f* ^2^≥0.15, and *f* ^2^≥0.35, respectively. All the statistical analyses were performed using python version 3.9.18 with the numpy, statsmodel, pandas, pingouin, seaborn, scipy and matplotlib libraries.

Statistical power was determined through post hoc power analysis using G*Power 3.1.9.6 [34]. Regarding the analyses involving the ADOS-2 CSS and ADI-R (subdomains A, B and C), with α = 0.05, a sample size of 127, a total of 3 predictors (clinical measure + age + sex) and an effect size of f² = 0.15, the calculated statistical power (1 - β) for the beta and gamma ROI analyses was 0.96. For the additional analyses conducted on the sensory-assessed subgroup of 57 participants, and under the same parameters, the statistical power was 0.65.

## Results

### Associations between beta/gamma powers and overall autistic symptomatology

We found no significant associations between beta or gamma power and overall symptomatology, based on the clinical data collected for this study (*n* = 127 for the ADOS-2 CSS and *n* = 116 for the SRS-2 Total T-score) (Supplementary Table S5).

### Associations between beta/gamma powers and the social domain

No significant relationships were found between beta or gamma power and social dimensions using ADI-R subdomains A and B (*n* = 127) and SRS-2 social subdomains (*n* = 113) (Supplementary Table S6).

### Associations between beta/gamma powers and sensory processing and RRBs

#### Correlations between beta power and sensory processing

The results showed a significant correlation between beta PSD and hyper- and hyposensory scores (Table 3).

**Table 3:** Beta and gamma powers correlations with repetitive and sensory measures across six regions

			*Frontal*	*Central*	*Parietal*	*Occipital*	*R. Temporal*	*L. Temporal*
**BETA**	**ADI-R C**	***R*** ^**2**^	0.007	0.006	0.005	0.01	0.03	0.03
		***f*** ^**2**^	0.007	0.006	0.005	0.01	0.03	0.03
		***p***	0.45	0.41	0.75	0.86	0.11	0.34
		***p***_***corr***_	0.675	0.675	0.86	0.86	0.66	0.675
	**SRS-2 RRB** **T-score**	***R*** ^**2**^	0.01	0.008	0.004	0.02	0.04	0.02
		***f*** ^**2**^	0.01	0.008	0.004	0.02	0.04	0.02
		***p***	0.34	0.43	0.72	0.74	0.48	0.66
		***p***_***corr***_	0.74	0.74	0.74	0.74	0.74	0.74
	**SSP** **Hyper**	***R*** ^**2**^	0.08	0.14	0.11	0.03	0.10	0.13
		***f*** ^**2**^	0.09	0.16	0.12	0.03	0.11	0.15
		***p***	**0.04**	**0.01**	**0.02**	0.32	0.05	**0.02**
		***p***_***corr***_	0.06	0.04*	0.04*	0.32	0.06	0.04*
	**SSP** **Hypo**	***R*** ^**2**^	0.12	0.26^†^	0.17	0.11	0.18	0.17
		***f*** ^**2**^	0.14	0.35^†^	0.20	0.12	0.22	0.20
		***p***	**0.01**	**0.0007** ^†^	**0.004**	**0.022**	**0.003**	**0.006**
		***p***_***corr***_	0.01*	0.004**	0.008**	0.02*	0.008**	0.009**
**GAMMA**	**ADI-R C**	***R*** ^**2**^	0.03	0.03	0.004	0.02	0.07	0.08
		***f*** ^**2**^	0.03	0.03	0.004	0.02	0.08	0.09
		***p***	0.16	0.38	0.60	0.91	**0.02**	0.16
		***p***_***corr***_	0.32	0.57	0.72	0.91	0.06	0.32
	**SRS-2 RRB** **T-score**	***R*** ^**2**^	0.03	0.06	0.01	0.02	0.08	0.04
		***f*** ^**2**^	0.03	0.06	0.01	0.02	0.09	0.04
		***p***	0.91	0.12	0.37	0.60	0.72	0.43
		***p***_***corr***_	0.91	0.72	0.86	0.86	0.86	0.86
	**SSP** **Hyper**	***R*** ^**2**^	0.04	0.08	0.05	0.02	0.02	0.09
		***f*** ^**2**^	0.04	0.09	0.05	0.02	0.02	0.10
		***p***	0.45	0.76	0.60	0.53	0.92	0.20
		***p***_***corr***_	0.90	0.91	0.90	0.90	0.92	0.90
	**SSP** **Hypo**	***R*** ^**2**^	0.03	0.08	0.04	0.01	0.03	0.05
		***f*** ^**2**^	0.03	0.09	0.04	0.01	0.03	5
		***p***	0.68	0.66	0.81	0.94	0.39	0.66
		***p***_***corr***_	0.94	0.94	0.94	0.94	0.94	0.94

A separate multiple linear regression model was run for each power frequency-clinical measure combination

Age and sex were included as covariates in the models. ^†^As the central beta power–hyposensitivity model violated the assumption of homoscedasticity, we applied robust standard errors (HC3) in the OLS model. Significant p-values are marked in bold and p-values remaining significant after FDR (False Discovery Rate) correction are marked with an asterisk (*p_corr_ < 0.05; **p_corr_ < 0.01)

*Abbreviations: ADI-R C* Autism Diagnostic Interview Revised, Stereotypes & restricted interests domain score, *SRS-2 RRB T-score* Social Responsiveness Scale, Second Edition, Restricted and Repetitive Behavior Standardized T-score, *SSP* Short Sensory Profile, Hyper-Hyposensory scores, *R* Right, *L* Left

**Fig. 1:**
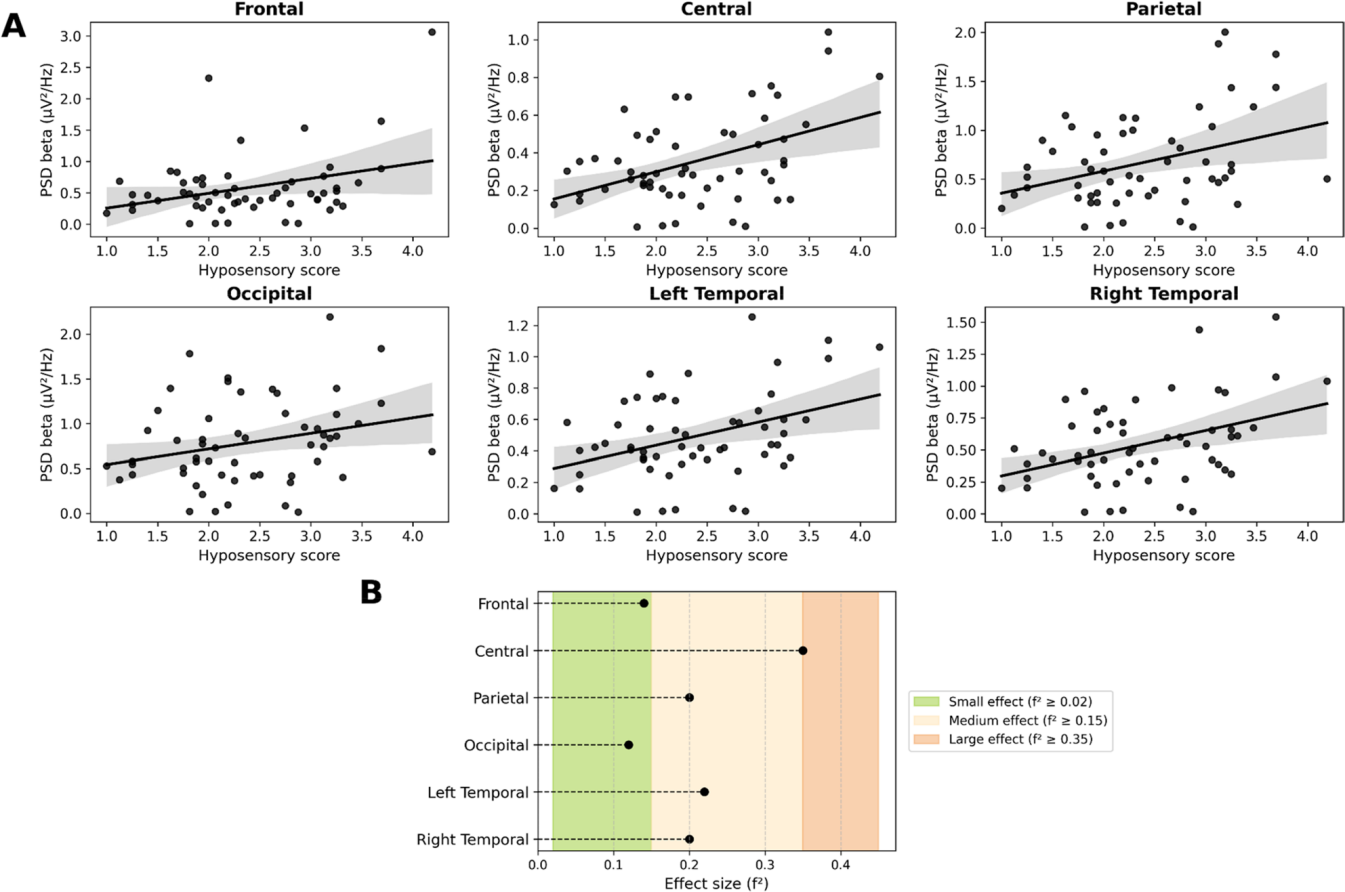
**A** Linear relationships of beta PSD (in µV²/Hz) as a function of the hyposensory score for the six regions of interest (ROIs), *n* = 57. **B** Effect size (f²) by ROI *PSD : Power Spectral Density*

Figure 1 shows a positive correlation between the hyposensory score and beta oscillatory power. This means that as the hyposensory score increases, beta power increases across all the ROIs (frontal R^2^ = 0.12, *f* ^2^=0.14, *p*_*corr*_=0.01, central R^2^ = 0.26, *f* ^2^=0.35, *p*_*corr*_=0.003, parietal R^2^ = 0.17, *f* ^2^=0.20, *p*_*corr*_=0.008, occipital R^2^ = 0.11, *f* ^2^=0.12, *p*_*corr*_=0.02, right temporal R^2^ = 0.18, *f* ^2^=0.22, *p*_*corr*_=0.008, left temporal R^2^ = 0.17, *f* ^2^=0.20, *p*_*corr*_=0.009) (Fig. 1A). Cohen’s *f* ^2^ between 0.15 and 0.35 (4/6) illustrates moderate to large effect sizes, while Cohen’s *f* ^2^ between 0.02 and 0.15 illustrates small effect sizes (2/6) (Table 3; Fig. 1B).

**Fig. 2:**
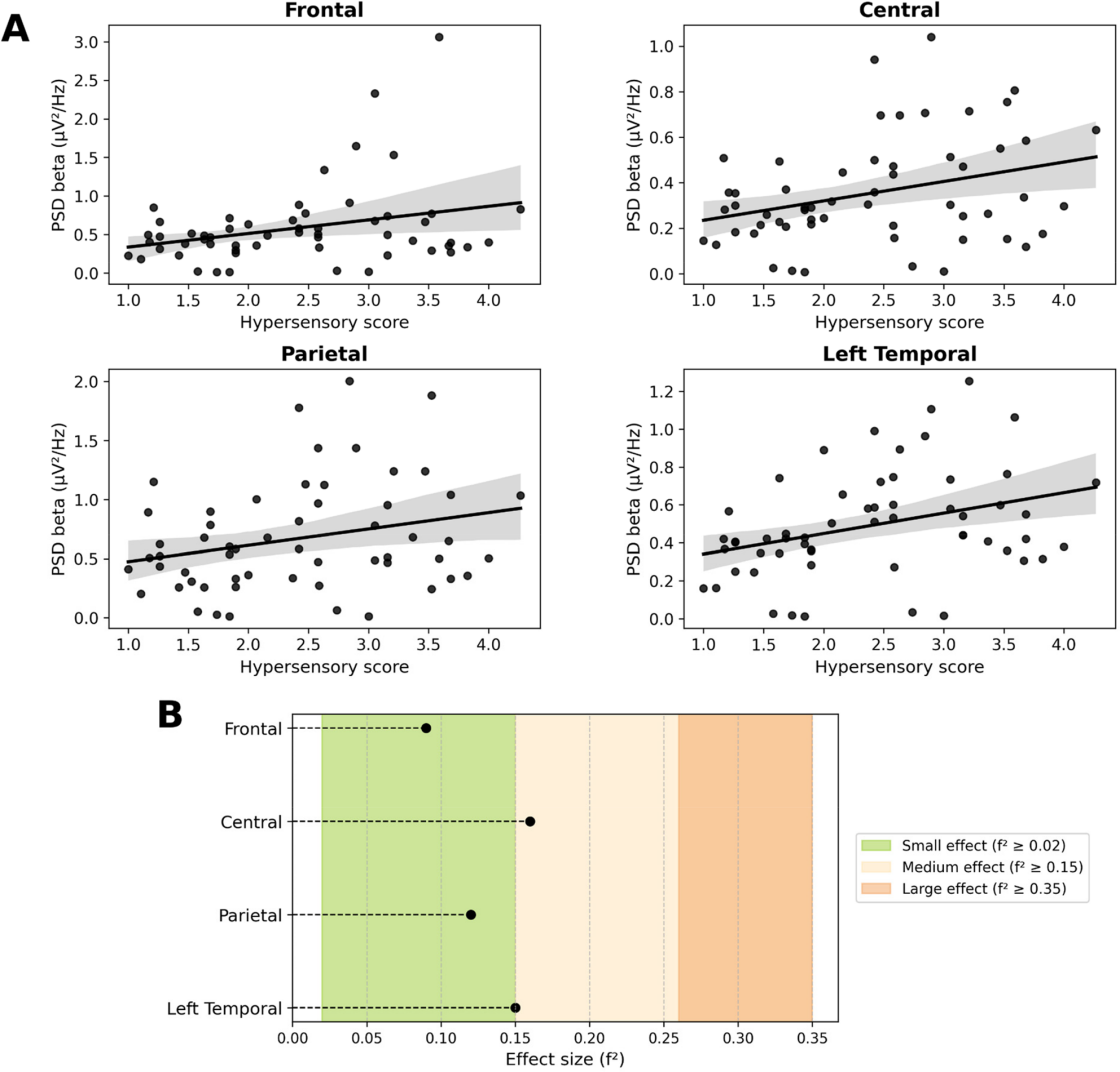
**A** Linear relationships of beta PSD (in µV²/Hz) as a function of the hypersensory score for the frontal, central, parietal and left temporal regions, *n* = 57. **B** Effect size (f²) by region of interest (ROI) *PSD : Power Spectral Density*

Figure 2 shows similar results for hypersensitivity. Indeed, the linear models indicate that as the hypersensory score increases, beta power also increases for the central (R^2^ = 0.14, *f* ^2^=0.16, *p*_*corr*_=0.04), parietal (R^2^ = 0.11, *f* ^2^=0.12, *p*_*corr*_=0.04) and left temporal (R^2^ = 0.13, *f* ^2^=0.15, *p*_*corr*_=0.04) regions. The frontal region also shows a positive correlation but does not survive the FDR correction (R^2^ = 0.08, *f* ^2^=0.09, *p* = .04, *p*_*corr*_=0.06). (Fig. 2A). Cohen’s *f* ^2^ values are between 0.09 and 0.16 (4/4), indicating small to moderate effect sizes (Table 3; Fig. 2B).

#### Correlations between gamma power and RRBs

**Fig. 3:**
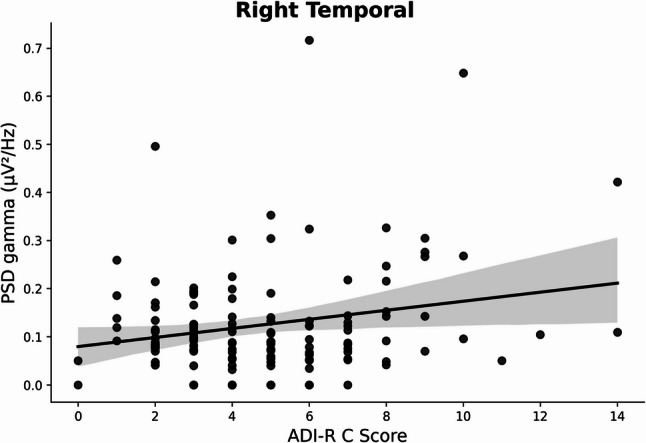
Linear relationship of gamma PSD (in µV²/Hz) as a function of the ADI-R C score for the right temporal region, *n* = 127 *PSD : Power Spectral Density*,* ADI-R C: Autism Diagnostic Interview Revised*,* Stereotypes & restricted interests domain score*

Without FDR correction, the results show a positive correlation (R^2^ = 0.07, *f*^2^ = 0.08, *p* = .02) (Fig. 3) between gamma oscillatory power in the right temporal region and the severity of RRB symptoms measured by the ADI-R C. However, this correlation did not survive after multiple comparisons correction (*p*_*corr*_=0.06) and Cohen’s f² value (0.08) illustrates a small effect size (Table 3).

## Discussion

Identifying biomarkers of ASD is a crucial challenge for enabling earlier diagnoses and for improving our understanding of the underlying physiological mechanisms. However, this task is complex because of the significant inter-individual phenotypic and endophenotypic variability, the limited number of subjects in most studies, and the predominant focus on categorical diagnostic frameworks with insufficient integration of dimensional data [10].

While dimensional approaches are now well established in ASD research, applying them to large, monocentric cohorts with homogeneous clinical assessments remains essential to improve interpretability and reproducibility. In this study, we addressed some of these challenges by leveraging the SoNeTAA platform to establish one of the largest monocentric cohorts of autistic persons, with standardized dimensional clinical data across a broad neurodevelopmental age range. All diagnoses were performed by the same experts, minimizing variability and strengthening the robustness of our findings.

When exploring the relationship between resting-state beta and gamma oscillatory powers and autistic symptomatology, we found relevant results between beta power and sensory processing, and exploratory results between gamma power and RRBs. Although we examined all the clinical components, our results focused specifically on two symptoms present in category B of the DSM-5 ASD diagnosis. In contrast, no significant associations were identified between beta or gamma power and category A symptoms involving social communication and interaction. This absence of association was consistent across all social communication measures, including the ADI-R subdomains A and B and the SRS-2 social subscales, possibly reflecting limited sensitivity of these tools to subtle variations in social domains. Overall, the pattern of results suggests domain-specific associations, preferentially involving sensory and repetitive dimensions rather than social communication deficits.

### Correlations between beta power and sensory processing

Sensory sensitivities, including both hyper- and hyposensitivity, are core features of ASD, observed up to 90% of autistic individuals [35, 36]. These sensory challenges are closely tied to the balance between excitatory and inhibitory neurotransmission, with GABA and glutamate playing crucial roles [37, 38]. Disruptions in this balance may contribute to the sensory symptoms observed in ASD, which often emerge early in development and affect all sensory domains.

Our study provided an innovative analysis using hyper- and hyposensory scores to better account for sensory variability. Their relationship with the E/I balance was investigated by examining the power of beta and gamma oscillations, which serve as indirect electrophysiological features reflecting network dynamics related to the E/I balance.

A significant and relevant correlation between beta power and hyper- and hyposensory scores in autistic subjects was observed in our results. High hyposensory scores were associated with increased beta power in the frontal, central, parietal, occipital, and temporal regions. Similarly, high hypersensory scores are also linked to increased beta power in the frontal, central, parietal, and temporal regions. These results suggest that sensory atypicalities in ASD may be related to changes in brain oscillatory activity. Notably, the relationship between sensory processing in ASD and the E/I balance has been demonstrated in the literature. For example, one study revealed that lower levels of GABA in the sensorimotor cortex of the brain are associated with abnormal tactile processing in children with ASD [38]. Moreover, other preclinical studies revealed that E/I balance disruptions in Shank3 mutant mice (a monogenic mouse model of autism) are linked to impaired sensory integration of auditory and tactile stimuli [39, 40]. Thus, an alteration of network dynamics related to the E/I balance, may result in poor integration of sensory signals, causing abnormal responses to sensory stimuli in individuals with ASD.

Hyposensory processing in ASD is rarely described in the literature; however, it is a common feature and appears in ASD during early childhood, making our results particularly noteworthy [30, 41, 42]. The hyper- and hyposensory scores used in this study are rarely replicated in the literature, but being derived from the SSP, they are accessible in various cohorts and could be further explored.

Furthermore, both sensory subtype scores were associated with a significant increase in beta power. Given that sensory hyper and hyposensitivity are not mutually exclusive and are both encompassed within the diagnostic criteria for ASD, this pattern likely reflects common difficulties in sensory regulation rather than distinct sensory profiles. Additional analyses revealed a correlation between these hypo- and hypersensitivity scores (Supplementary Figure S8). From a clinical perspective, it is interesting to note that individuals with ASD can exhibit both sensory features simultaneously, even within the same sensory domain. These findings suggest a potential alteration in sensory filtering, where overload in one sensory modality could reduce the ability to process others. Some hypotheses propose local hyperconnectivity, which amplifies responses to certain sensory stimuli, and long-range hypoconnectivity between brain regions, preventing effective regulation across sensory modalities and leading to multisensory integration difficulties.

While some studies have reported region-specific alterations, particularly in sensorimotor cortices [38], these findings often rely on task-based paradigms or more focal source-level analyses [43]. In contrast, resting-state scalp-level EEG captures broader network-level dynamics [44], which may account for the more global patterns observed here.

### Correlations between gamma power and RRBs

No significant associations emerged between gamma power and restricted and repetitive behaviors after correction, despite a trend-level link observed in the right temporal region. Gamma oscillations play a crucial role in synchronizing neural networks involved in cognitive and sensory information processing and have been described as indirect features reflecting network dynamics related to the E/I balance [12, 13]. An anomaly in this rhythm could disrupt how the brain processes such information. Moreover, the human brain shows hemispheric asymmetry and the right temporal region is often linked to cognitive and socio-emotional functions [45]. Alterations in this region have been reported in ASD and are associated with aberrant brain growth and overconnectivity [46, 47]. Therefore, an alteration in gamma oscillations in the right temporal region may indicate disruptions in how socio-emotional, cognitive, and sensory information is processed and integrated. These disruptions in information processing could contribute to the RRBs observed in individuals with ASD.

This gamma-power trend should be interpreted with caution, as scalp EEG is poorly suited to high-frequency activity, highly susceptible to artifacts and low signal-to-noise ratio and is typically low-pass filtered around 50 Hz [48]. These methodological limitations likely reduced sensitivity and contributed to the lack of significant associations after correction. MEG could be more robust and better suited for this type of high-frequency analysis [49].

### Link between RRBs and sensoriality in ASD

The previous results support the idea that an increase in beta power is associated with sensory atypicalities, and that an increase in gamma power may be linked to more severe RRBs. The connection between these two ASD symptoms has been well-documented in the scientific literature. Correlational studies have shown that children with high levels of repetitive behaviors also exhibit high levels of sensory atypicality [50–52]. Additionally, sensory integration-based interventions indicate that children with ASD receiving therapies to improve sensory processing also experience a reduction in repetitive behaviors [53, 54]. These two symptoms are classified within the same diagnostic cluster, making their association appear logical and their characteristics difficult to distinguish.

As previously mentioned, RRBs may be partly due to altered sensory information processing. Some authors suggest that this alteration could lead to hypersensitivity, potentially increasing anxiety, which may drive individuals with ASD to engage in repetitive behaviors to create a sense of predictability and control [55, 56]. Conversely, these atypicalities could result in hyposensitivity, prompting the search for additional sensory stimulation through repetitive behaviors. Thus, RRBs may help regulate sensory experiences and reduce anxiety in autistic individuals. Taken together, these findings partially suggest that RRBs and sensory atypicalities may share a common neurobiological basis, potentially involving an imbalance in the E/I balance.

### Limitations

The limitations of this study include several factors that may influence the results. First, a subset of participants was receiving pharmacological treatment at the time of EEG recording, which may modulate neural activity. The heterogeneity and small size of treated subgroups prevented a full characterization of medication effects. Conversely, excluding these treated participants would have removed individuals with potentially more severe clinical profiles, thereby reducing the sample’s representativeness. Exploratory analyses were therefore conducted to assess the effect of medication use (Supplementary Tables S1–S2). Additionally, our sample exhibits a marked male predominance (~ 7:1), exceeding the commonly reported male-to-female ratio of approximately 3:1 [57]. We hypothesize that this discrepancy may reflect characteristics of the French healthcare system, whereby more severe clinical presentations (more frequently observed in males) are preferentially referred to hospital-based services, while milder forms (more often identified in females) are typically managed in outpatient or community-based settings. However, it is crucial to consider sex differences in EEG profiles, as noted in some studies [58–60] and in our complementary analyses (Supplementary Figure S9). This sex imbalance in our dataset may therefore limit the generalizability of the findings, particularly to female autistic individuals.

The clinical context of data collection led to missing values and made it difficult to obtain data in a homogeneous and standardized manner. Other scales specifically targeting repetitive behaviors, such as the Repetitive Behavior Scale-Revised (RBS-R), could have provided a more detailed assessment of these symptoms [61]. However, this scale could not be included in our dataset because of the extent of missing data. In addition, an important limitation must be considered regarding the results for RRBs. Domain C of the ADI-R includes item 71, which assesses sensory processing in individuals. However, these data were not available in our dataset, preventing us from verifying whether our results were influenced by this item.

This study does not include a comparison with a neurotypical group, as the objective was to examine intra-group variations in individuals with ASD. However, as previously mentioned, the literature reports differences in beta and gamma powers between individuals with ASD and controls [11, 22].

### Perspectives

Although our current results are based on resting-state data, future studies including sensory tasks, such as Mismatch Negativity and vibrotactile paradigms, will allow us to explore beta power dynamics during active sensory processing. In addition, using a single method and biomarker provides a partial view of the E/I balance. Thus, this work will be expanded with a more comprehensive approach incorporating additional electrophysiological proxy markers described in the literature through EEG (e.g. aperiodic 1/f signal, neural entropy) [12], with the aim of integrating them into predictive models to better characterize network-level dynamics related to E/I balance. In addition, future studies should examine oscillatory activity across the full frequency spectrum to provide a more comprehensive understanding of these dynamics.

From a methodological perspective, future work could further refine our approach by developing normative models that account for neurodevelopmental trajectories, particularly within our 5- to 17-year-old cohort, either by leveraging larger reference datasets or by analyzing data by age group. In parallel, exploring automated preprocessing methods and comparing them with manual inspection could help evaluate potential differences in outcomes and assess the most effective approach for artifact rejection. Finally, our analysis focused on the eyes-closed condition to ensure signal consistency and comparability with previous ASD studies [62]. This may, however, limit generalization to other resting-state contexts, and future work should include eyes-open recordings for comparison.

Although the analyses were conducted on a sample of autistic participants, our findings on sensory processing might reflect variability that also exists in the general population. Further studies could help clarify the role of beta power in typical sensory integration.

## Conclusion

In conclusion, these results further support the growing body of evidence indicating that research on ASD biomarkers must rigorously account for its neurobiological diversity and individual differences to reveal clinically relevant distinctions. The findings of this study highlighted a significant link between beta power and atypical sensory processing in individuals with ASD, while exploratory analyses suggested a potential association between gamma power and restricted and repetitive behaviors. To our knowledge, this is the first study in a clinical model to demonstrate a significant link between beta oscillatory power and sensory processing.

## Supplementary Information

Supplementary Material 1.

## Data Availability

The data that support the findings of this study are available on request from the corresponding author. The data are not publicly available due to privacy or ethical restrictions. The code used for the analysis is publicly available at https://github.com/juliechaudet/PSD-Analysis-EEG-HR.

## Abbreviations

ADI-R: Autism Diagnostic Interview - Revised
ADOS-2: Autism Diagnostic Observation Schedule - Second Edition
ASD: Autism Spectrum Disorder
DSM–5: Diagnostic and Statistical Manual of Mental Disorders, 5th edition
E/I: Excitation/Inhibition
FDR: False Discovery Rate
HD-EEG: High-Density Electroencephalography
PSD: Power Spectral Density
RRBs: Restricted and Repetitive Behaviors
ROI: Region of Interest
SRS-2: Social Responsiveness Scale, Second Edition
SSP: Short Sensory Profile
