# Supplementary material for: Beta power as a neural correlate of sensory features in autistic individuals
Source: J Neurodev Disord. 2026 Mar 18;18:23. doi: 10.1186/s11689-026-09685-1 (PMC13147585; doi:10.1186/s11689-026-09685-1)
Supplement: Supplementary file 1 — Supplementary Material 1. [file 11689_2026_9685_MOESM1_ESM.docx]

**Supplementary materials**

**Beta power as a neural correlate of sensory features in autistic individuals**

Julie Chaudet ^1,2^ , Julien Pichot ^3^, Amandine Pedoux ^4^ , Mathis Fleury ^3^,Anna Maruani ^4^ ,Valérie Vantalon ^4^, Elise Humeau ^4^, Thomas Bourgeron ^3^, Josselin Houenou ^1,5^ , Guillaume Dumas ^6^ ,Edouard Duchesnay ^2^, Richard Delorme ^3,4^, Anton Iftimovici ^7,8^, Aline Lefebvre ^1,9,10^

^1^ NeuroSpin, UNIACT Unit, PsyBrain Team CEA Paris-Saclay, Gif-Sur-Yvette, France

^2^ NeuroSpin, BAOBAB Unit, GAIA Team, CEA Paris-Saclay, Gif-Sur-Yvette, France

^3^ Institut Pasteur, Université Paris Cité, ‘Human Genetics and Cognitive Functions’ Team, CNRS UMR 3571, Paris, France

^4^ Child and Adolescent Psychiatry Department, Robert Debré University Hospital, APHP, and Université Paris Cité, Paris, France.

^5^ Department of Psychiatry, Faculty of Medicine, Mondor University Hospital, APHP, ‘Translational Psychiatry’ Team 15, INSERM U955, Créteil, France.

^6^ Department of Psychiatry, Faculty of Medicine, CHU Sainte-Justine Research Centre, ‘Precision Psychiatry and Social Physiology’ Team, Université de Montréal, Montréal, QC, Canada.

^7^ Institute of Psychiatry and Neuroscience of Paris (IPNP), Université Paris Cité, INSERM U1266, ‘Pathophysiology of psychiatric disorders’ Team, CNRS GDR 3557, Paris, France

^8^ GHU Paris Psychiatrie et Neurosciences, Sainte Anne Hospital, APHP, Paris, France

^9^ Faculty of Medicine, Université Paris-Saclay, Kremlin-Bicêtre, France

^10^ Neurodevelopmental Disorders Advice and Expertise Service, Fondation Vallée, Gentilly, France

**Supplementary Table S1.** Medication profiles by group category.

|  |  | **Complete**  **group** | **SRS-assessed subgroup** | **Sensory-assessed**  **subgroup** |
| --- | --- | --- | --- | --- |
|  |  | Number of participants (N) | | |
| **All participants** | | 127 | 116 | 57 |
| **Untreated participants** | | 83 | 76 | 35 |
| **Treated participants** | | 44 | 40 | 22 |
| **Melatonin** | All | 13 | 13 | 9 |
|  | Melatonin | 10 | 10 | 6 |
|  | Prolonged-release | 3 | 3 | 3 |
| **Second Generation antipsychotic (SGA)** | All | 8 | 8 | 3 |
|  | Abilify | 3 | 3 | 1 |
|  | Risperdal | 5 | 5 | 2 |
| **Methylphenidate** | All | 16 | 14 | 7 |
|  | Ritalin | 11 | 10 | 5 |
|  | Quasym | 5 | 4 | 2 |
| **Multiple*** | All | 7 | 5 | 3 |

*Multiple : Participants receiving more than one medication

*Untreated participants : Participants without medication*

*Treated participants : Participant with a single medication*

*Complete group : All participants in the sample*

*Sensory-assessed subgroup : Subgroup of participants with an assessment of sensory symptoms and the associated clinical scores*

*SRS-assessed subgroup : Subgroup of participants with an assessment of the SRS and the associated clinical scores*

**Supplementary Table S2.** Effect of medication use on beta and gamma powers in the complete group and sensory-assessed subgroup.

|  |  |  | **Frontal** | | **Central** | | **Parietal** | | **Occipital** | | **Temporal Left** | | **Temporal Right** | |
| --- | --- | --- | --- | --- | --- | --- | --- | --- | --- | --- | --- | --- | --- | --- |
|  |  |  | *Beta* | *Gamma* | *Beta* | *Gamma* | *Beta* | *Gamma* | *Beta* | *Gamma* | *Beta* | *Gamma* | *Beta* | *Gamma* |
| **Medication**  **effect**  **Any vs None** | Complete group | **F** | .08 | 1.4 | 1.2 | 3.7 | 1.4 | 1.4 | 2.73 | 1.2 | .60 | 1.5 | .32 | .80 |
|  |  | ***p*** | .77 | .23 | .26 | .06 | .23 | .24 | .10 | .30 | .44 | .22 | .57 | .36 |
|  |  | **η²** | .001 | .01 | .01 | .03 | .01 | .01 | 0.2 | .01 | .005 | .01 | .003 | .007 |
|  | Sensory- assessed  subgroup | **F** | 2.15 | 1.6 | .18 | 1.4 | 1.8 | .27 | 3.16 | 1.1 | .06 | 1.2 | .000 | .90 |
|  |  | ***p*** | .15 | .21 | .67 | .24 | .18 | .60 | .08 | .30 | .80 | .30 | .98 | .35 |
|  |  | **η²** | .04 | .03 | .003 | .03 | .03 | .005 | .06 | .02 | .001 | .02 | .000 | .02 |
|  |  |  |  |  |  |  |  |  |  |  |  |  |  |  |
| **Medication**  **type effect** | Complete group | **F** | .54 | 1.7 | 1.1 | 1.8 | 1.16 | 1.6 | 1.46 | 1.3 | 1.4 | 1.1 | 1.4 | 1.6 |
|  |  | ***p*** | .70 | .15 | .36 | .12 | .33 | .18 | .22 | .30 | .23 | .37 | .23 | .17 |
|  |  | **η²** | .02 | .05 | .03 | .06 | .04 | .05 | .05 | .04 | .204 | .03 | .05 | .05 |
|  | Sensory- assessed  subgroup | **F** | 1.9 | 1.3 | 1.13 | 1.2 | 1.15 | 1.2 | 1.9 | 1.5 | 2.5 | .90 | 2.11 | 1.0 |
|  |  | ***p*** | .12 | .30 | .35 | .36 | .34 | .33 | .12 | .20 | .06 | .50 | .10 | .42 |
|  |  | **η²** | .13 | .10 | .08 | .08 | .08 | .09 | .13 | .11 | .16 | .06 | .14 | .07 |

*The upper panel reports ANCOVA results comparing treated participants to untreated participants (controlling for age and sex), and the lower panel reports ANCOVA results showing the effect of medication type, comparing the group of untreated participants with groups treated with melatonin, SGA, methylphenidate, or multiple medications, while controlling for age and sex. Results are presented for both the full sample (Complete group, n=127) and the subsample with sensory scores (Sensory-assessed subgroup, n=57). p > .05 indicates no difference in beta and gamma powers between groups.*

**Supplementary Table S3.** Organization of hyper- and hyposensitivity scores from the 38-item Short Sensory Profile (SSP).

| **TACTILE SENSITIVITY** | | |
| --- | --- | --- |
| **No.** | **Item Description** | **Type** |
| **1** | Expresses distress during grooming (for example, fights or cries during haircut, face washing, fingernail cutting) | **HYPER** |
| **2** | Prefers long-sleeved clothing when it is warm or short sleeves when it is cold | **HYPO** |
| **3** | Avoids going barefoot, especially in sand or grass | **HYPER** |
| **4** | Reacts emotionally or aggressively to touch | **HYPER** |
| **5** | Withdraws from splashing water | **HYPER** |
| **6** | Has difficulty standing in line or close to other people | **HYPER** |
| **7** | Rubs or scratches out a spot that has been touched | **BOTH** |
|  |  |  |
| **TASTE & SMELL SENSITIVITY** | | |
| **No.** | **Item Description** | **Type** |
| **8** | Avoids certain tastes or food smells that are typically part of children's diets | **HYPER** |
| **9** | Will only eat certain tastes | **HYPER** |
| **10** | Limits self to particular food textures/temperatures | **HYPER** |
| **11** | Picky eater, especially regarding food textures | **HYPER** |
|  |  |  |
| **MOTION SENSITIVITY** | | |
| **No.** | **Item Description** | **Type** |
| **12** | Becomes anxious or distressed when feet leave the ground | **HYPER** |
| **13** | Fears or avoids heights | **BOTH** |
| **14** | Dislikes activities where head is upside down | **HYPER** |
|  |  |  |
| **HYPOREACTIVITY / SENSATION-SEEKING** | | |
| **No.** | **Item Description** | **Type** |
| **15** | Enjoys strange noises/seeks to make noise for noise's sake | **HYPO** |
| **16** | Seeks all kinds of movement and this interferes with daily routines | **HYPO** |
| **17** | Becomes easily excitable during movement activity | **HYPO** |
| **18** | Touches people and objects | **HYPO** |
| **19** | Doesn't seem to notice when face or hands are messy | **HYPO** |
| **20** | Jumps from one activity to another so that it interferes with play | **HYPO** |
| **21** | Leaves clothing twisted on body | **HYPO** |
|  |  |  |
| **AUDITORY FILTERING** | | |
| **No.** | **Item Description** | **Type** |
| **22** | Is distracted or has trouble functioning if there is a lot of noise around | **HYPER** |
| **23** | Appears to not hear what you say | **HYPO** |
| **24** | Can't work with background noise | **HYPER** |
| **25** | Has trouble completing tasks when the radio is on | **HYPER** |
| **26** | Doesn't respond when name is called but you know the child's hearing is OK | **HYPO** |
| **27** | Has difficulty paying attention | **BOTH** |
|  |  |  |
| **LOW ENERGY** | | |
| **No.** | **Item Description** | **Type** |
| **28** | Seems to have weak muscles | **HYPO** |
| **29** | Slumps, bumps, especially when standing or holding particular body position | **HYPO** |
| **30** | Has a weak grasp | **HYPO** |
| **31** | Can't lift heavy objects | **HYPO** |
| **32** | Props to support self | **HYPO** |
| **33** | Poor endurance/tires easily | **HYPO** |
|  |  |  |
| **VISUAL SENSITIVITY** | | |
| **No.** | **Item Description** | **Type** |
| **34** | Responds negatively to unexpected or loud noises | **HYPER** |
| **35** | Holds hands over ears to protect ears from sound | **HYPER** |
| **36** | Is bothered by bright lights after others have adapted to the light | **HYPER** |
| **37** | Watches everyone when they move around the room | **HYPER** |
| **38** | Covers eyes or squints to protect eyes from light | **HYPER** |

Table inspired by Lefebvre et al., (2023) Tackling hypo and hyper sensory processing heterogeneity in autism: From clinical stratification to genetic pathways. *Autism research*, 16(2), 364–378. <https://doi.org/10.1002/aur.2861>

**Supplementary table S4.** Influence of individual observations on linear models relating beta PSD to hyper and hyposensory scores and gamma PSD to ADI-R C score.

|  |  |  |  | **Complete model** | | | **Model without the influential point** | | |
| --- | --- | --- | --- | --- | --- | --- | --- | --- | --- |
| **HYPO** | **Influential point index** | **Region** | **Cook D** | ***p*** | ***R*^2^** | ***f* ^2^** | ***p*** | ***R*^2^** | ***f* ^2^** |
|  | 10 | *Central* | .17 | .0007 | .26 | .35 | .0002 | .26 | .35 |
|  |  | *Parietal* | .09 | .004 | .17 | .20 | .003 | .18 | .22 |
|  |  | *Temporal Left* | .09 | .006 | .17 | .20 | .005 | .17 | .20 |
|  | 27 | *Frontal* | .74 | .01 | .12 | .14 | .02 | .11 | .13 |
|  |  | *Parietal* | .08 | .004 | .17 | .20 | .003 | .17 | .21 |
|  | 42 | *Central* | .12 | .0007 | .26 | .35 | .001 | .23 | .30 |
|  |  | *Parietal* | .10 | .004 | .17 | .20 | .007 | .17 | .20 |
|  |  | *Occipital* | .09 | .02 | .11 | .12 | .040 | .10 | .11 |
|  |  | *Temporal Left* | .08 | .006 | .17 | .20 | .010 | .15 | .17 |
|  | 43 | *Frontal* | .12 | .01 | .12 | .14 | .02 | .11 | .12 |
|  |  | *Central* | .24 | .0007 | .26 | .35 | .003 | .20 | .24 |
|  |  | *Temporal Left* | .12 | .006 | .17 | .20 | .02 | .15 | .17 |
|  |  | *Temporal Right* | .33 | .003 | .18 | .22 | .01 | .15 | .17 |
|  |  |  |  |  |  |  |  |  |  |
| **HYPER** | 27 | *Frontal* | .17 | .04 | .08 | .09 | .02 | .10 | .11 |
|  | 43 | *Frontal* | .14 | .04 | .08 | .09 | .04 | .09 | .10 |
|  |  | *Central* | .27 | .01 | .14 | .16 | .02 | .14 | .17 |
|  |  | *Parietal* | .10 | .02 | .11 | .12 | .03 | .10 | .11 |
|  |  | *Temporal Left* | .15 | .02 | .13 | .15 | .02 | .15 | .17 |
|  |  |  |  |  |  |  |  |  |  |
| **ADI-R C** | 104 | *Temporal Right* | .09 | .02 | .07 | .08 | .03 | .07 | .08 |

*Influence diagnostics for individual observations on linear models relating beta-band PSD to hyposensory (HYPO, n=57) and hypersensory (HYPER, n=57) scores and gamma-band PSD to ADI‑R C scores (n=127). Observations on the Cook’s distance plots (Supplementary Figures S5-S7) were selected for influence analysis if they were identified as influential in at least three regions and/or showed a Cook’s distance greater than twice the adjusted threshold (D > 2 × 0.075 ≈ 0.15). For each selected point and region, the table reports Cook’s distance, p‑values, R^2^ and effect size f^2^ for the complete model and for the model refitted after exclusion of that influential observation.*

*A p < .05 in the model recalculated without the influential point indicates that this point is not sufficiently decisive to alter the significance of the initial effect.*

*PSD : Power Spectral Density, ADI-R C: Autism Diagnostic Interview Revised, Stereotypes & restricted interests domain score.*

**Supplementary Table S5.** Beta and gamma powers correlations with overall autistic clinical assessment measures across six regions.

|  |  |  | *Frontal* | *Central* | *Parietal* | *Occipital* | *R. Temporal* | *L. Temporal* |
| --- | --- | --- | --- | --- | --- | --- | --- | --- |
| **BETA** | **SRS-2**  **total**  **T-score** | ***R*^2^** | .009 | .004 | .01 | .01 | .02 | .03 |
|  |  | ***f* ^2^** | .009 | .004 | .01 | .01 | .02 | .03 |
|  |  | ***p*** | .47 | .54 | .38 | .82 | .92 | .89 |
|  |  | ***p_corr_*** | .93 | .93 | .93 | .93 | .51 | .42 |
|  | **ADOS-2**  **CSS** | ***R*^2^** | .008 | .001 | .007 | .01 | .01 | .02 |
|  |  | ***f* ^2^** | .008 | .001 | .007 | .01 | .01 | .02 |
|  |  | ***p*** | .42 | .71 | .58 | .76 | .60 | .76 |
|  |  | ***p_corr_*** | .76 | .76 | .76 | .76 | .76 | .76 |
|  |  |  |  |  |  |  |  |  |
| **GAMMA** | **SRS-2**  **total**  **T-score** | ***R*^2^** | .02 | .03 | .002 | .02 | .03 | .07 |
|  |  | ***f* ^2^** | .02 | .03 | .002 | .02 | .03 | .08 |
|  |  | ***p*** | .39 | .52 | .90 | .68 | .90 | .67 |
|  |  | ***p_corr_*** | .90 | .90 | .90 | .90 | .90 | .90 |
|  | **ADOS-2**  **CSS** | ***R*^2^** | .05 | .04 | .002 | .02 | .05 | .08 |
|  |  | ***f* ^2^** | .05 | .04 | .002 | .02 | .05 | .09 |
|  |  | ***p*** | .05 | .34 | .84 | .48 | .08 | .20 |
|  |  | ***p_corr_*** | .24 | .51 | .84 | .57 | .24 | .40 |

*A separate multiple linear regression model was run for each power frequency-clinical measure combination. Age and sex were included as covariates in the models. Significant p-values are marked in bold and p-values remaining significant after FDR (False Discovery Rate) correction are marked with an asterisk (*p_corr_ < .05; **p_corr_ < .01).*

*Abbreviations: ADOS-2 CSS: Autism Diagnostic Observation Schedule—second version—Calibrated Severity Score ; SRS-2 total T-score: Social Responsiveness Scale, Second Edition, total Standardized T-score; R: Right, L: Left.*

**Supplementary Table S6.** Beta and gamma powers correlations with social clinical assessment measures across six regions.

|  |  |  | *Frontal* | *Central* | *Parietal* | *Occipital* | *R. Temporal* | *L. Temporal* |
| --- | --- | --- | --- | --- | --- | --- | --- | --- |
| **BETA** | **SRS-2**  **Social motivation**  **T-score** | ***R*^2^** | .004 | .004 | .01 | .02 | .04 | .03 |
|  |  | ***f* ^2^** | .004 | .004 | .01 | .02 | .04 | .03 |
|  |  | ***p*** | .75 | .60 | .33 | .55 | .70 | .70 |
|  |  | ***p_corr_*** | .75 | .75 | .75 | .75 | .75 | .75 |
|  | **SRS-2**  **Social communication**  **T-score** | ***R*^2^** | .02 | .002 | .007 | .01 | .03 | .02 |
|  |  | ***f* ^2^** | .02 | .002 | .007 | .01 | .03 | .02 |
|  |  | ***p*** | .23 | .65 | .98 | .75 | .50 | .51 |
|  |  | ***p_corr_*** | .90 | .90 | .98 | .90 | .90 | .90 |
|  | **SRS-2**  **Social awareness**  **T-score** | ***R*^2^** | .006 | .002 | .008 | .02 | .04 | .02 |
|  |  | ***f* ^2^** | .006 | .002 | .008 | .02 | .04 | .02 |
|  |  | ***p*** | .67 | .67 | .65 | .42 | .62 | .50 |
|  |  | ***p_corr_*** | .67 | .67 | .67 | .67 | .67 | .67 |
|  | **SRS-2**  **Social cognition**  **T-score** | ***R*^2^** | .02 | .01 | .01 | .01 | .06 | .05 |
|  |  | ***f* ^2^** | .02 | .01 | .01 | .01 | .06 | .05 |
|  |  | ***p*** | .21 | .30 | .50 | .76 | .14 | .10 |
|  |  | ***p_corr_*** | .42 | .45 | .06 | .76 | .42 | .42 |
|  | **ADI-R**  **A** | ***R*^2^** | .005 | .001 | .01 | .03 | .03 | .02 |
|  |  | ***f* ^2^** | .005 | .001 | .01 | .03 | .03 | .02 |
|  |  | ***p*** | .58 | .73 | .40 | .16 | .16 | .66 |
|  |  | ***p_corr_*** | .87 | .87 | .87 | .87 | .87 | .87 |
|  | **ADI-R**  **B** | ***R*^2^** | .02 | .03 | .03 | .02 | .02 | .04 |
|  |  | ***f* ^2^** | .02 | .03 | .03 | .02 | .02 | .04 |
|  |  | ***p*** | .17 | .06 | .08 | .21 | .32 | .12 |
|  |  | ***p_corr_*** | .25 | .24 | .24 | .25 | .32 | .24 |

| **GAMMA** | **SRS-2**  **Social motivation**  **T-score** | ***R*^2^** | .03 | .04 | .006 | .02 | .08 | .03 |
| --- | --- | --- | --- | --- | --- | --- | --- | --- |
|  |  | ***f* ^2^** | .03 | .04 | .006 | .02 | .09 | .03 |
|  |  | ***p*** | .53 | .72 | .64 | .84 | .86 | .93 |
|  |  | ***p_corr_*** | .93 | .93 | .93 | .93 | .93 | .93 |
|  | **SRS-2**  **Social communication**  **T-score** | ***R*^2^** | .03 | .04 | .007 | .02 | .07 | .03 |
|  |  | ***f* ^2^** | .03 | .04 | .007 | .02 | .07 | .03 |
|  |  | ***p*** | .40 | .70 | .97 | .75 | .57 | .85 |
|  |  | ***p_corr_*** | .97 | .97 | .97 | .97 | .97 | .97 |
|  | **SRS-2**  **Social awareness**  **T-score** | ***R*^2^** | .03 | .04 | .01 | .03 | .08 | .03 |
|  |  | ***f* ^2^** | .03 | .04 | .03 | .03 | .09 | .03 |
|  |  | ***p*** | .80 | .45 | .24 | .22 | .73 | .91 |
|  |  | ***p_corr_*** | .90 | .90 | .72 | .72 | .90 | .90 |
|  | **SRS-2**  **Social cognition**  **T-score** | ***R*^2^** | .06 | .04 | .006 | .02 | .10 | .05 |
|  |  | ***f* ^2^** | .06 | .04 | .006 | .02 | .11 | .05 |
|  |  | ***p*** | .06 | .99 | .58 | .87 | .17 | .23 |
|  |  | ***p_corr_*** | .36 | .99 | .87 | .99 | .46 | .46 |
|  | **ADI-R**  **A** | ***R*^2^** | .01 | .03 | .01 | .03 | .03 | .07 |
|  |  | ***f* ^2^** | .01 | .03 | .01 | .03 | .03 | .08 |
|  |  | ***p*** | .83 | .93 | .20 | .22 | .36 | .32 |
|  |  | ***p_corr_*** | .93 | .93 | .54 | .54 | .54 | .54 |
|  | **ADI-R**  **B** | ***R*^2^** | .02 | .04 | .02 | .02 | .03 | .07 |
|  |  | ***f* ^2^** | .02 | .04 | .02 | .02 | .03 | .09 |
|  |  | ***p*** | .60 | .33 | .13 | .67 | .36 | .64 |
|  |  | ***p_corr_*** | .67 | .67 | .67 | .67 | .67 | .67 |

*A separate multiple linear regression model was run for each power frequency-clinical measure combination. Age and sex were included as covariates in the models. Significant p-values are marked in bold and p-values remaining significant after FDR (False Discovery Rate) correction are marked with an asterisk (*p_corr_ < .05; **p_corr_ < .01).*

*Abbreviations: SRS-2 T-score: Social Responsiveness Scale, Second Edition, Standardized T-score; ADI-R: Autism Diagnostic Interview Revised; ADI-R A: ADI-R Social interaction domain score; ADI-R B: ADI-R Communication domain score; R: Right, L : Left.*


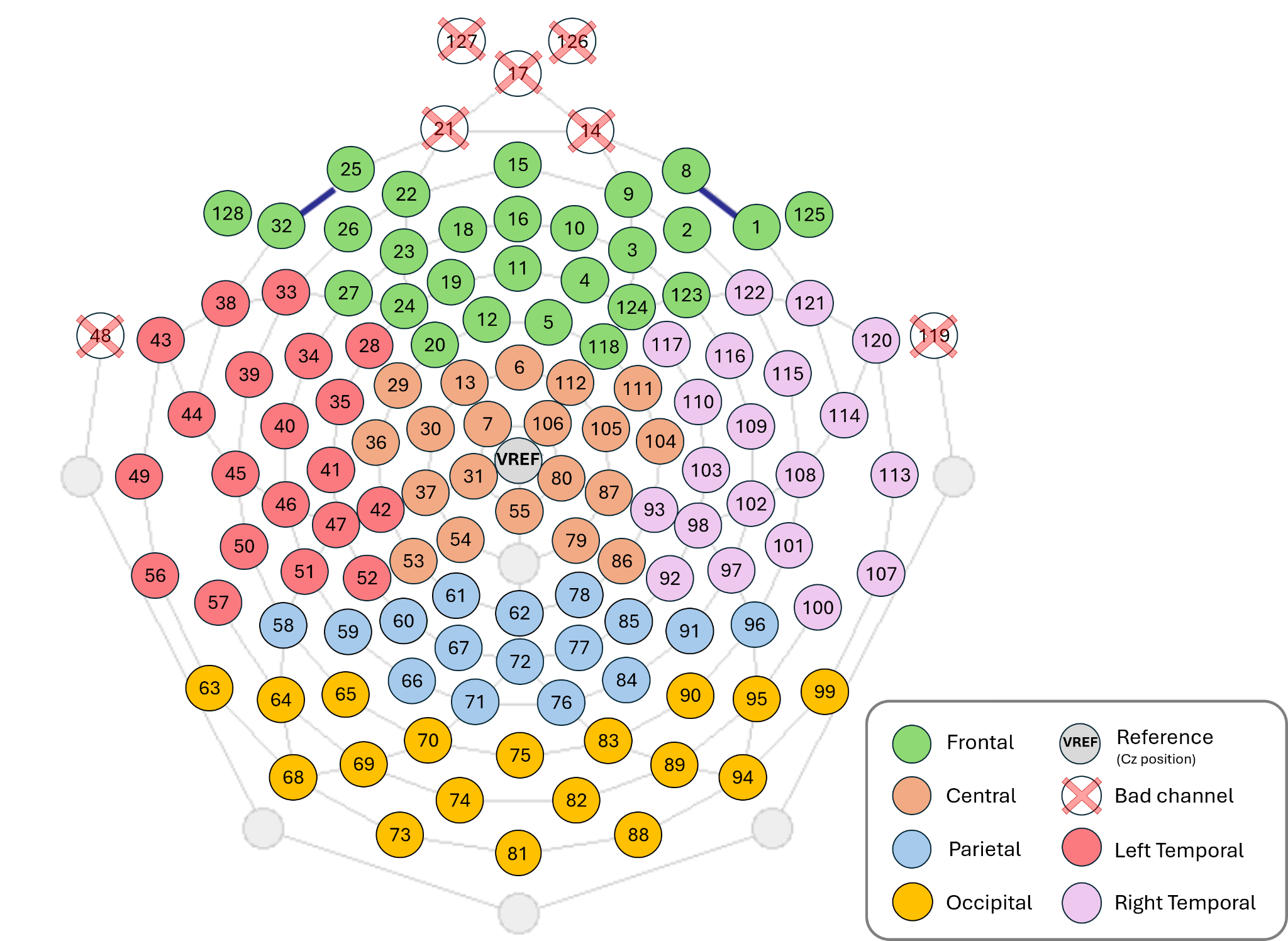


**Supplementary Figure S1.** Organisation of the EEG montage into six regions of interest : frontal (channels 1,2,3,4,5, 8,9,10,11,12, 15,16,18,19,20,22, 23, 24, 25, 26, 27, 32, 118, 123, 124, 125, 128), central (channels 6, 7, 13, 29, 30, 31, 36, 37, 53, 54, 55, 79, 80, 86, 87, 104, 105, 106, 111, 112), parietal (channels 58, 59, 60, 61, 62, 66, 67, 71, 72, 76, 77, 78, 84, 85, 91, 96), occipital (channels 63, 64, 65, 68, 69, 70, 73, 74, 75, 81, 82, 83, 88, 89, 90, 94, 95, 99), left temporal (channels 28, 33, 34, 35, 38, 39, 40, 41, 42, 43, 44, 45, 46, 47, 49, 50, 51, 52, 56, 57) and right temporal (channels 92, 93, 97, 98, 100, 101, 102, 103, 107, 108, 109, 110, 113, 114, 115, 116, 117, 120, 121, 122).


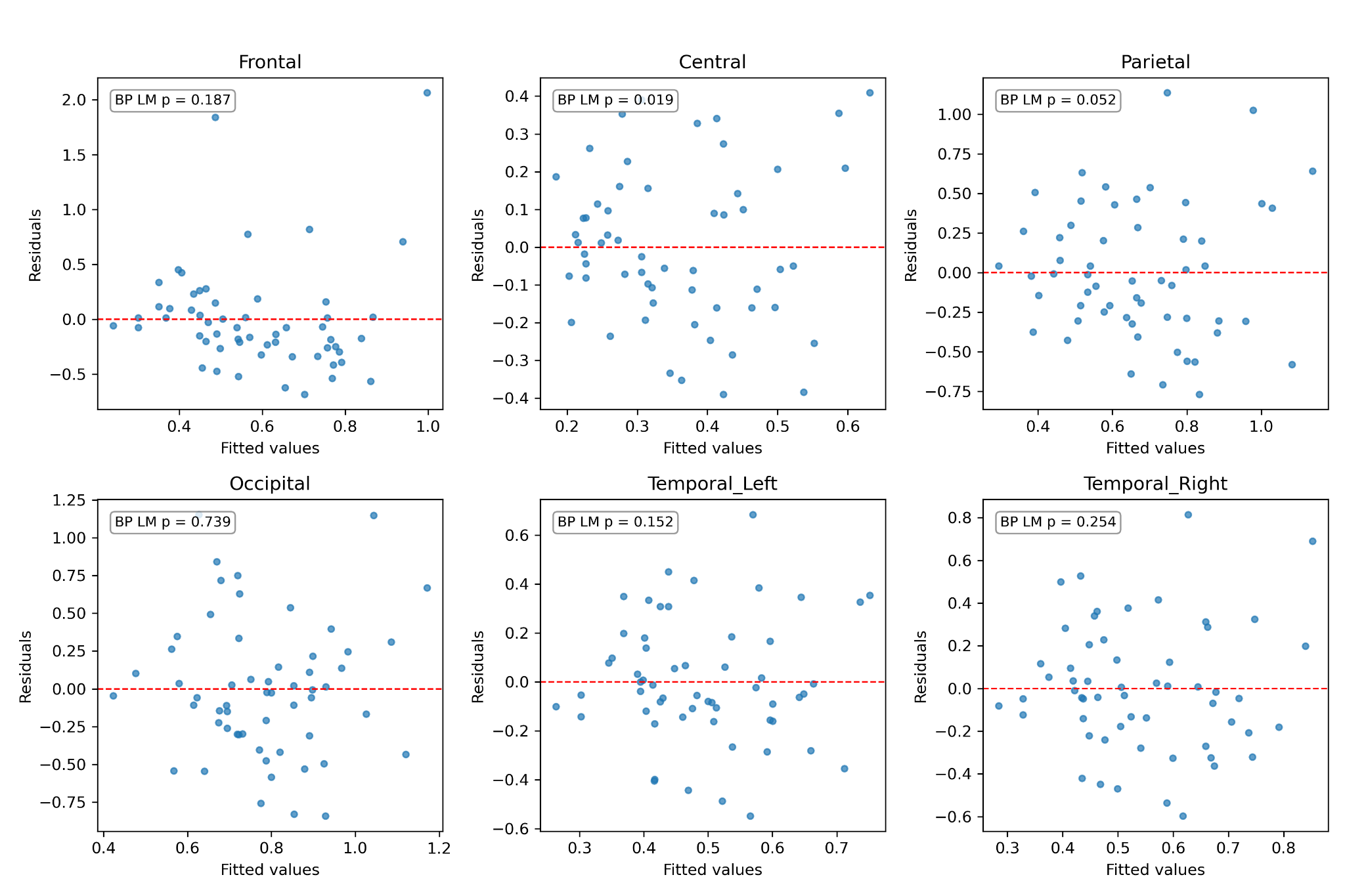
**Supplementary Figure S2.** Residuals vs fitted plots for linear models relating beta PSD to hyposensory scores.

*Each panel displays a residuals vs fitted plot for the linear regression model relating beta PSD to hyposensory scores in each region of interest (n=57). The p-value of the Breusch–Pagan test is reported in each panel; p > .05 indicates no evidence of heteroscedasticity for the corresponding model.*

*PSD : Power Spectral Density*


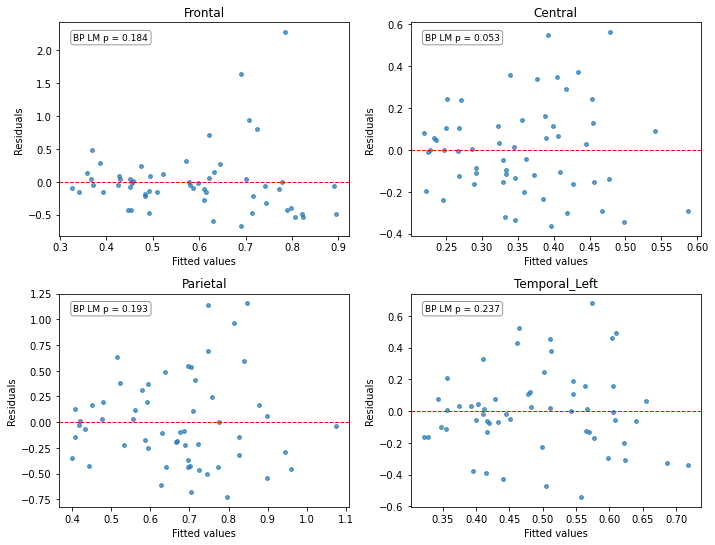


**Supplementary Figure S3.** Residuals vs fitted plots for linear models relating beta PSD to hypersensory scores.

*Each panel displays a residuals vs fitted plot for the linear regression model relating beta PSD to hypersensory scores in frontal, central, parietal and left temporal regions (n=57). The p-value of the Breusch–Pagan test is reported in each panel; p > .05 indicates no evidence of heteroscedasticity for the corresponding model.*

*PSD : Power Spectral Density*


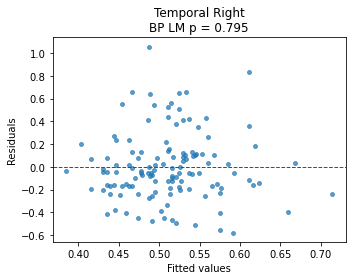


**Supplementary Figure S4.** Residuals vs fitted plots for linear model relating gamma PSD to ADI-R C scores in temporal right region.

*Residuals vs fitted plot for the linear regression model relating beta PSD to ADI-R C scores in temporal right region (n=127). The p-value of the Breusch–Pagan test is reported; p > .05 indicates no evidence of heteroscedasticity for this model.*

*PSD : Power Spectral Density, ADI-R C: Autism Diagnostic Interview Revised, Stereotypes & restricted interests domain score.*


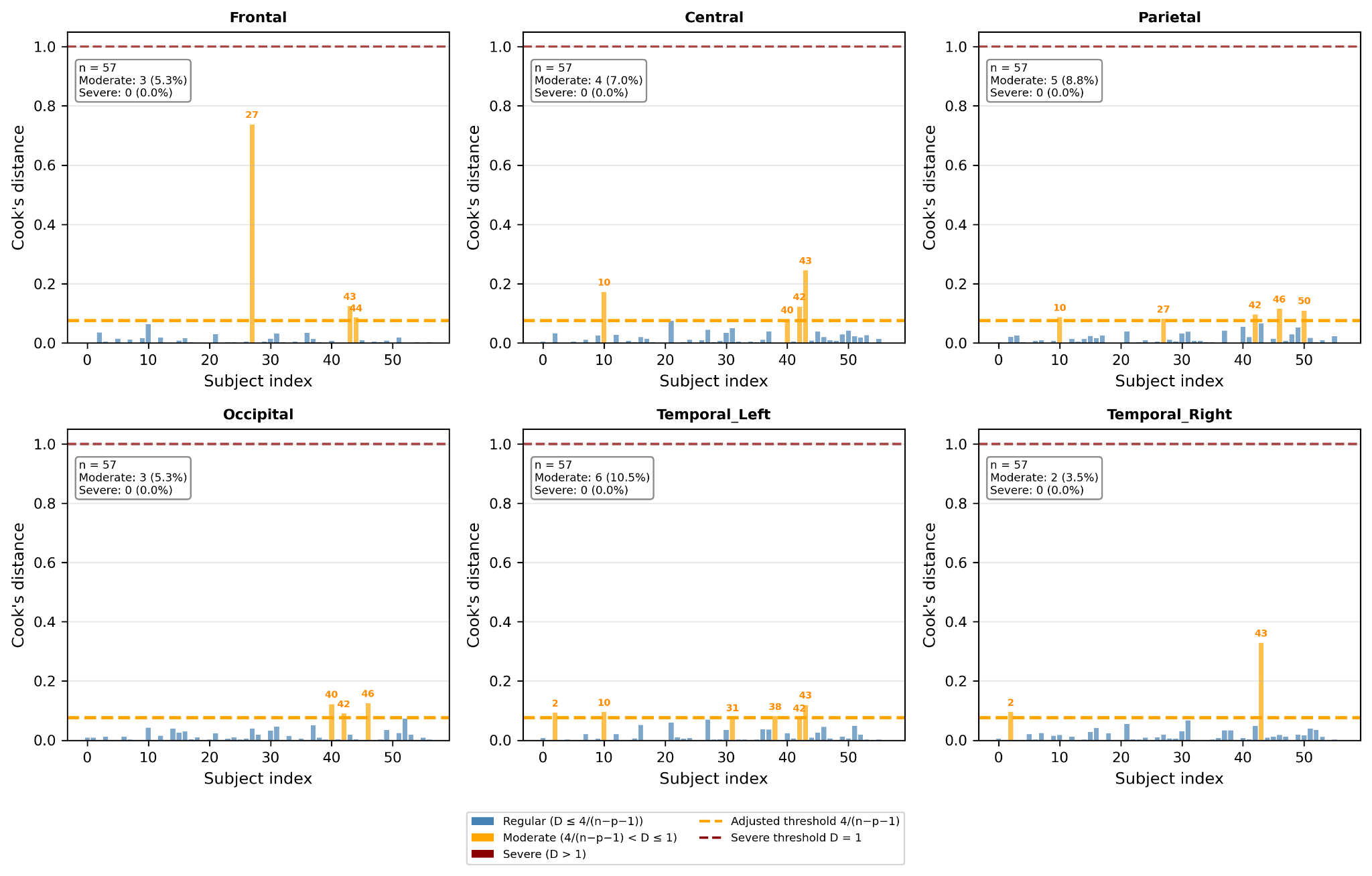


**Supplementary Figure S5.** Cook’s distance influences plots for linear models relating beta PSD to hyposensory scores.

*The graphs present the distribution of Cook's distances for each subject (n = 57) across six regions of interest. Blue bars represent individual Cook's distance values for each subject, with the following thresholds: orange dashed line = adjusted threshold (4/(n-p-1) < D ≤ 1), red dashed line = severe threshold (D > 1). Subjects with values above the adjusted threshold are identified by their number. Insets indicate the total number of subjects as well as the proportion of moderate and severe observations detected in each region. No severe observations (D > 1) were detected across all analyzed regions.*

*PSD : Power Spectral Density*


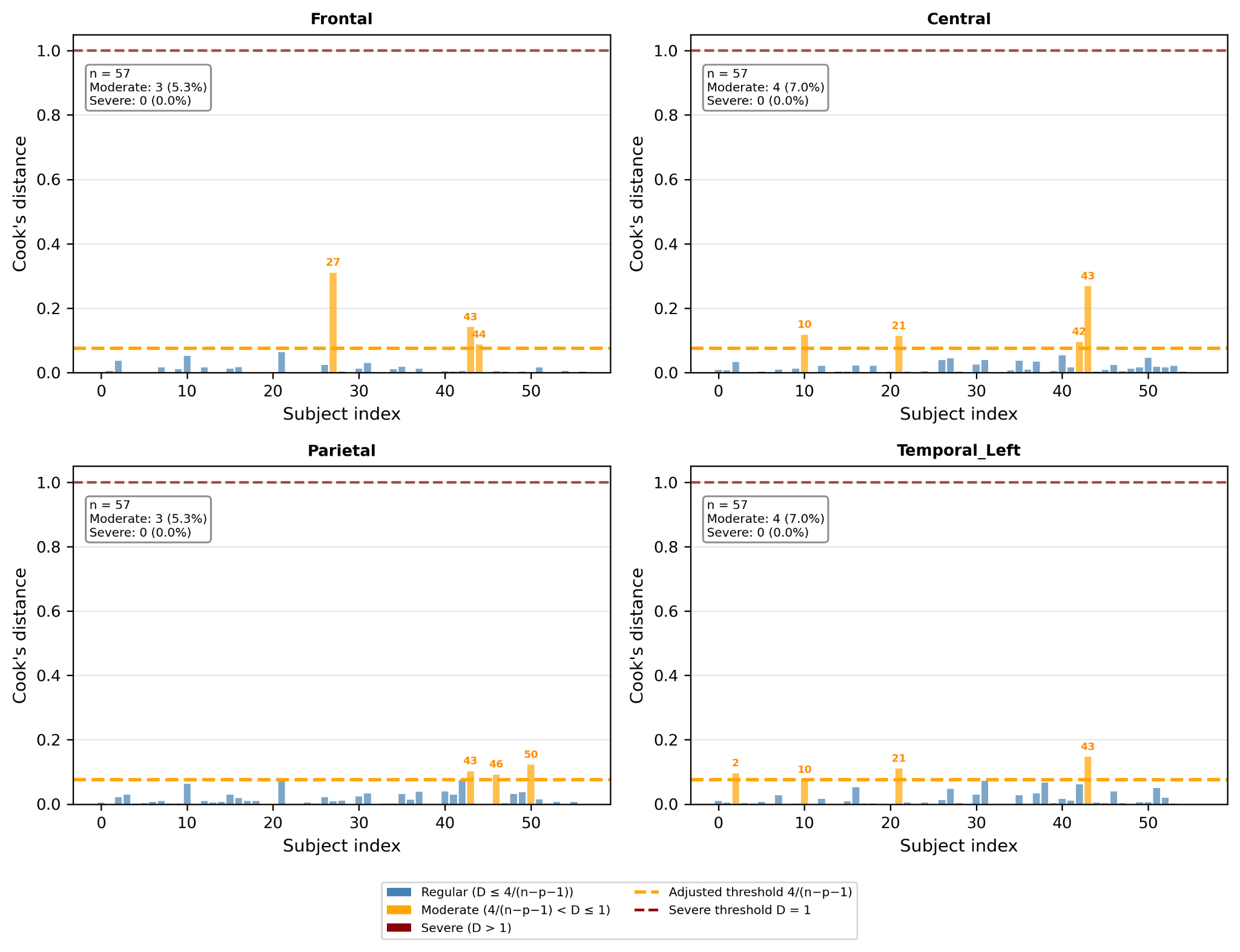


**Supplementary Figure S6.** Cook’s distance influences plots for linear models relating beta PSD to hypersensory scores.

*The graphs present the distribution of Cook's distances for each subject (n = 57) for frontal, central, parietal and left temporal regions. Blue bars represent individual Cook's distance values for each subject, with the following thresholds: orange dashed line = adjusted threshold (4/(n-p-1) < D ≤ 1), red dashed line = severe threshold (D > 1). Subjects with values above the adjusted threshold are identified by their number. Insets indicate the total number of subjects as well as the proportion of moderate and severe observations detected in each region. No severe observations (D > 1) were detected across all analyzed regions.*

*PSD : Power Spectral Density*


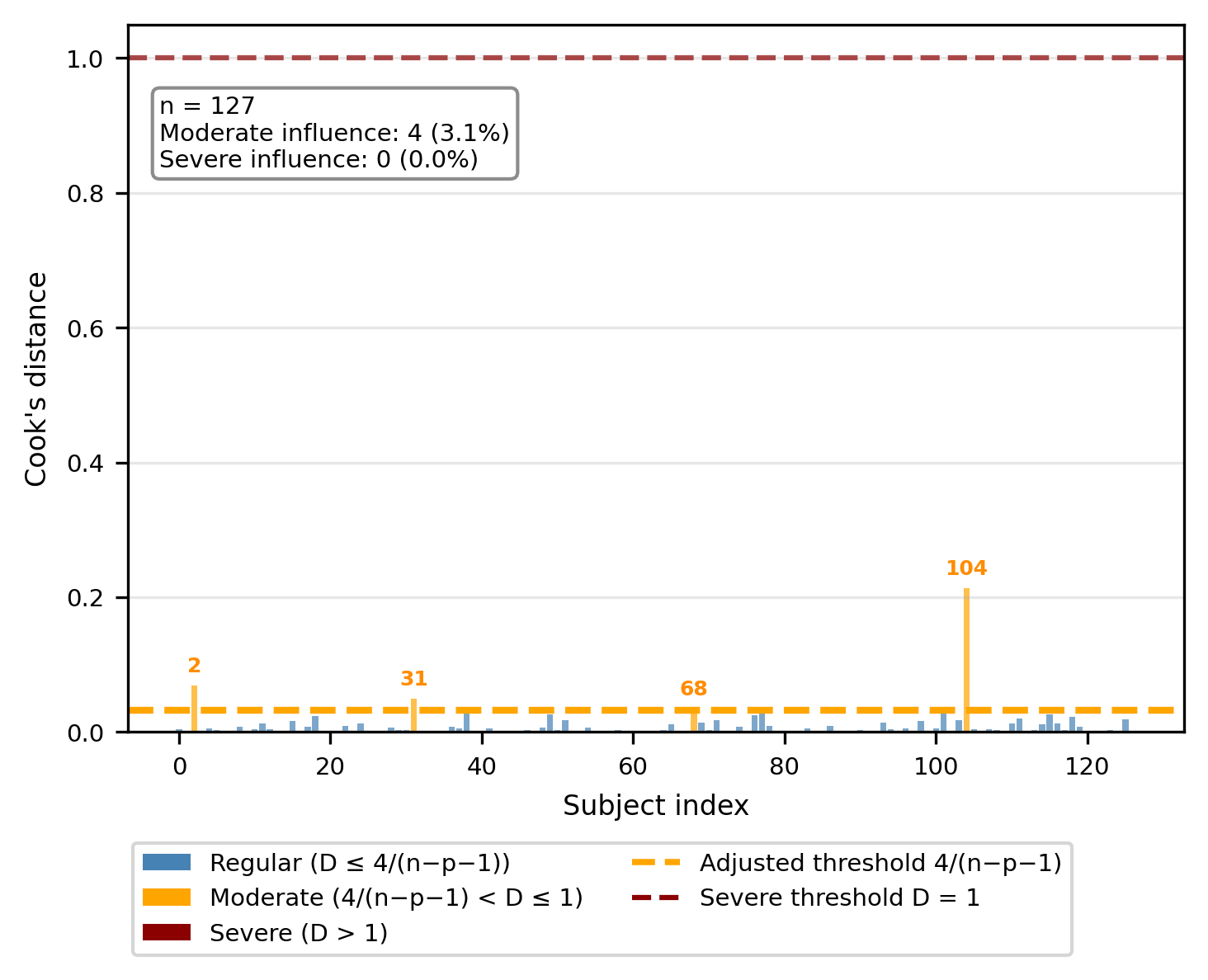


**Supplementary Figure S7.** Cook’s distance influence plot for the linear model relating to gamma PSD ADI-R C scores in the temporal right region.

*The graphs present the distribution of Cook's distances for each subject (n = 127) for the right temporal region. Blue bars represent individual Cook's distance values for each subject, with the following thresholds: orange dashed line = adjusted threshold (4/(n-p-1) < D ≤ 1), red dashed line = severe threshold (D > 1). Subjects with values above the adjusted threshold are identified by their number. Insets indicate the total number of subjects as well as the proportion of moderate and severe observations detected in each region. No severe observations (D > 1) were detected.*

*PSD : Power Spectral Density, ADI-R C: Autism Diagnostic Interview Revised, Stereotypes & restricted interests domain score.*


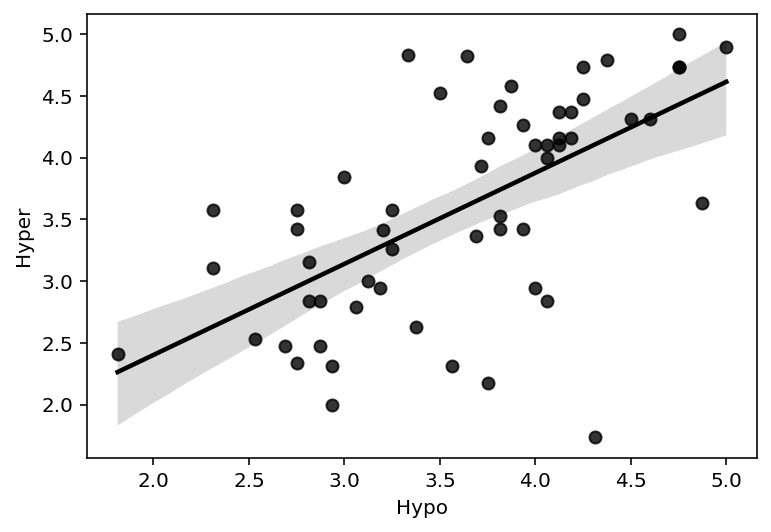


**Supplementary Figure S8.** Relationship between hyper- and hyposensory scores, n=57, *p*=4.36 e^-07^

**C.**


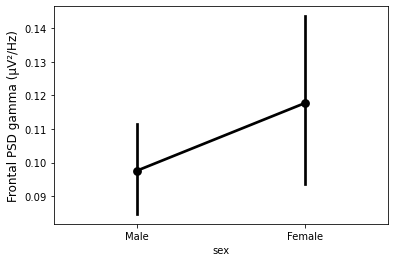

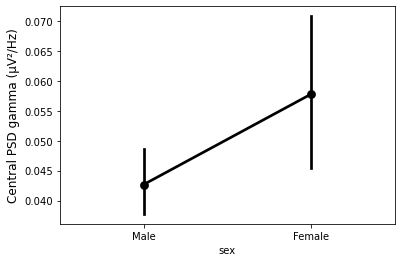


*****

**A.**

**B.**

| *  * | **Male** | **Female** |
| --- | --- | --- |
| **N**  **Total = 127** | 110 | 17 |
| **Age (years)**  Mean (SD) | 10.2  (3) | 10.5  (2.1) |
|  |  |  |
|  | **Beta**  **(*p _uncorr_)*** | **Gamma**  **(*p _uncorr_*)** |
| *Frontal* | 0.7 | **0.03** |
| *Central* | 0.7 | **0.01** |
| *Parietal* | 0.8 | 0.7 |
| *Occipital* | 0.2 | 0.15 |
| *R. Temporal* | 0.9 | 0.8 |
| *L. Temporal* | 0.8 | 0.6 |

**Supplementary Figure S9. A.** Descriptive information on males and females on the sample. **B.** Uncorrected p-values from the Mann-Whitney test comparing beta and gamma powers between sexes across the six ROIs. **C.** Graphical representation of significant results for gamma PSD differences between males and females in the frontal and central regions.
